# Sleep apnea and autonomic dysfunction in patients with dementia

**DOI:** 10.3389/fnins.2022.951147

**Published:** 2022-11-03

**Authors:** Michelle Herberts, Bhanuprakash Kolla, Travis Paul, Praveen Mekala, Meghna P. Mansukhani

**Affiliations:** ^1^Center for Sleep Medicine, Mayo Clinic, Rochester, MN, United States; ^2^Mayo Clinic Health System, Southwest Minnesota, Mankato, MN, United States

**Keywords:** obstructive sleep apnea, autonomic dysfunction, dementia, REM sleep behavior disorder, autonomic control

## Abstract

Sleep apnea is common sleep disorder that is associated with an is an increase in risk of many health conditions, including systemic hypertension, stroke, atrial fibrillation, and heart failure. The predominant underlying pathophysiological mechanism for elevated risk of these conditions in patients with sleep apnea is thought to involve autonomic dysfunction in the form of sympathetic overactivity. Autonomic dysfunction is also associated with several neurodegenerative disorders and sleep apnea, in turn, has been shown to be associated with an increased risk of development of mild cognitive impairment and various types of dementia. Rapid eye movement sleep behavior disorder, which is also associated with an increased risk of alpha synucleiopathy-related dementia, is also linked with autonomic dysfunction. In this article we explore the relationship between sleep apnea, autonomic dysfunction, rapid eye movement sleep behavior disorder and dementia. This article describes the various autonomic dysfunction that are thought to occur in the context of sleep apnea. And illustrate the mechanisms by which sleep apnea, through its impact on autonomic dysfunction could potentially result in dementia. We also review the evidence examining the impact of treatment of sleep apnea on autonomic dysfunction and cognitive outcomes.

## Introduction

Sleep apnea is a very common condition, with a prevalence of 10–17% in males and 3–9% in females, with overall prevalence continuing to increase throughout all age groups (Culebras and Anwar, 2018). Despite an increase in incidence and awareness, it is estimated that as many as 80% of cases go undiagnosed (Culebras and Anwar, 2018). OSA is generally defined as an apnea-hypopnea index (AHI) of greater than 5 events per hour on polysomnography or home sleep apnea testing (Gammoudi et al., 2015). Complete (apnea) and partial (hypopnea) disordered breathing events in sleep usually lead to arousals as well as changes in autonomic functions (Lombardi et al., 2018). Mild, moderate, and severe sleep apnea are defined as a respiratory disturbance index (RDI) of 5–14, 15–29, and ≥30, respectively. Prior studies have shown a link between sleep apnea and other cardiovascular conditions, including systemic hypertension, stroke, atrial fibrillation, and heart failure, with a 3-11-fold increase in these conditions if left untreated (Khoo and Chalacheva, 2016; Milagro et al., 2019; Dunietz et al., 2021).

Sleep apnea has also been shown to be associated with the development of cognitive decline and dementia through multiple potential mechanisms (Peter-Derex et al., 2015). It is postulated that presence of sleep apnea may lead to early emergence of dementia symptoms due to significant daytime somnolence (Mansukhani et al., 2019). Further, neurovascular complications associated with untreated sleep apnea may play a role in the development of cognitive dysfunction and vascular dementia (Culebras and Anwar, 2018; Mansukhani et al., 2019). Finally, there is a possibility that hypoxia related neuron dysfunction may play a part in the development of dementia (McCarter et al., 2020).

There is also an important relationship between the development of rapid eye movement sleep behavior disorder (RBD), cognitive decline, and autonomic function. RBD is a precursor for the development of alpha-synucleinopathies, such as Parkinson’s disease and dementia with Lewy bodies (Barone and Henchcliffe, 2018). Derangements in autonomic function have been described in patients with these disorders (Mansukhani et al., 2015; Barone and Henchcliffe, 2018). The relationship between these disorders and autonomic dysfunction is complex but is potentially thought to be due to the degeneration of autonomic fibers in the brainstem (Mansukhani et al., 2015).

**FIGURE 1:**
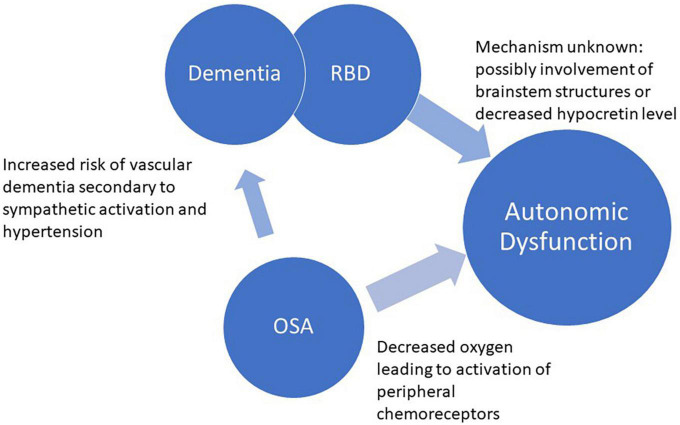
Relationship between OSA, autonomic dysfunction, RBD, and dementia.

This review article will describe the relationship between sleep apnea and autonomic control, autonomic dysfunction in RBD and dementia, as well as the possible associations between sleep apnea and dementia (Figure 1). We will first delineate the relationship between sleep apnea and its effects on the autonomic functions in the body. Then, we will describe the current literature assessing the link between autonomic dysfunction found in RBD and dementia. Lastly, we will review the current literature assessing the connection between sleep apnea and dementia.

## Sleep disorders and autonomic control

There is a complex relationship between autonomic function and sleep (Pepin et al., 2014; Miglis, 2016). In normal sleep, changes in heart rate and blood pressure are related to sleep stage. In non-rapid eye movement sleep, there is increased parasympathetic tone and decreased sympathetic tone which leads to decrease in heart rate, blood pressure, peripheral vascular resistance, and cardiac output. In rapid eye movement (REM) sleep, there is decreased ventilation and increased sympathetic activity with intermittent increases in heart rate and blood pressure (Del Rio et al., 2010; Silvani and Dampney, 2013; Miglis, 2016). The heart rate and blood pressure readings are similar to waking hours, however (Miglis, 2016). The chemoreflexes, particularly in the peripheral carotid sinus and carotid body, and autonomic functions have been shown to be deranged in patients with sleep apnea, both during sleep and wakefulness (Kara et al., 2003).

Moderate to severe obstructive sleep apnea (OSA) has been shown to be prevalent in 17% of middle-aged men and 9% of middle-aged women (Culebras and Anwar, 2018). OSA has been thought to lead to increased adverse cardiovascular outcomes, due in part to sympathetic overactivity (Somers et al., 1995). During apnea, forced inspiration against a closed glottis leads to hypoxia which contributes to activation of the autonomic nervous system via activation of peripheral chemoreceptors in the carotid sinus (Mansukhani et al., 2014). Activation of these chemoreceptors leads to increased blood pressure and peripheral vasoconstriction as a result of the sympathetic response (Endeshaw et al., 2009). Studies have shown that, during apnea, patients have swings in blood pressure and heart rate associated with sympathetic activation (Somers et al., 1995; Martínez-García et al., 2013; Miglis, 2016).

Vasoconstriction associated with ongoing sympathetic activation can sometimes be severe, leading to very high blood pressures, with more severe elevations corresponding to more severe and prolonged apneas (McCarter et al., 2020). In patients without OSA, blood pressure usually drops by approximately 10% lower during sleep due to decreased sympathetic tone (Somers et al., 1995; Lombardi et al., 2018). Patients with OSA have been shown to have absent nocturnal dipping of blood pressure, which indicates ongoing increase in sympathetic activation and tone, and is associated with increased risk of cardiovascular mortality (Narkiewicz et al., 1998; Lombardi et al., 2018). Interestingly, OSA has been shown to be present in 40–50% of patients with systemic hypertension and 90% of patients with resistant hypertension (Smith and Pacchia, 2007). Patients with resistant hypertension have been shown to be at increased risk of adverse cardiovascular events as well (Lombardi et al., 2018; Brunetti et al., 2019).

Sympathetic activation associated with apneas has been shown to persist during waking hours (Somers et al., 1995; Miglis, 2016; Gottlieb et al., 2017). If very severe, the hypoxia can also lead to drastic changes in heart rate control, sometimes leading to bradyarrhythmias, and even fatal arrhythmias (Somers et al., 1995; Miglis, 2016). Interestingly, intermittent hypoxia, whether acute or chronic, has been shown to demonstrate a more potent sympathetic response than acute sustained hypoxia (Espinar-Sierra et al., 1997; Martínez-García et al., 2013; Miglis, 2016). This has been thought to be due to an increase in the level of catecholamines in the circulation during intermittent hypoxia (Nashed et al., 2012; Martínez-García et al., 2013).

Treatment of OSA has been shown to reduce sympathetic activation and hypertensive episodes. One cohort showed that continuous positive airway pressure (CPAP) treatment increased parasympathetic activity after one week of treatment (Cohen et al., 2018). There has also been evidence of decreased sympathetic nervous activity in a few randomized control trials (Huynh et al., 2006; Marthol et al., 2006). Patients who were initiated on CPAP therapy were shown to have a decrease in diastolic and mean blood pressure when compared to controls, although data is mixed (Dauvilliers et al., 2012; Cohen et al., 2018; Brunetti et al., 2019). Blood pressure has been shown to be significantly reduced after at least 6 months of therapy, with patients with the highest number of hours of nocturnal CPAP usage achieving the most benefit. CPAP therapy has also been shown to improve cardiac parameters such as overall contractility and stroke volume after 6–8 weeks of treatment (Sieminski et al., 2017; Cohen et al., 2018).

Autonomic function has also been shown to be disrupted in other neurologic conditions as well. In a prospective review by Brunetti et al. (2019), patients with right-sided ischemic strokes were found to have significant alterations in autonomic nervous system (ANS) control. These patients were found to have an increase in mean heart rate during non-REM sleep. There was also found to be a reduction in low frequency sleep during REM, indicating decreased sympathetic tone. These changes were not evident in this cohort during non-REM sleep, indicating alterations in ANS control during the different sleep stages (Grimaldi et al., 2012). In addition, patients with Huntington’s disease were also noted to have higher average heart rate in wake and non-REM sleep but no change in REM sleep.

Autonomic dysfunction has also been shown to be more prevalent in patients with periodic limb movement disorder and restless legs syndrome, thought to be multifactorial in nature, leading to hypertension and vascular injury (St Louis and Boeve, 2017; Ashraf-Ganjouei et al., 2021). Sleep related bruxism and narcolepsy have also been associated with hypertension (Postuma et al., 2015; Dauvilliers et al., 2018). Sleep related hypertension secondary to bruxism is thought to be due to ongoing microarousals, leading to increased sympathetic activity (Lee et al., 2016; Postuma et al., 2019). Hypertension in narcolepsy patients is due to unknown causes but may be due to ongoing sleep arousals or deficiency in orexin, leading to changes in autonomic regulation (Ferini-Strambi et al., 2005; Kolla and Mansukhani, 2014; Barone et al., 2020).

## Autonomic dysfunction in rapid eye movement sleep behavior disorder and dementia

Rapid eye movement sleep behavior disorder (RBD) is characterized by incomplete or absent muscle paralysis during REM sleep, thus leading to forceful movements, vocalization, and dream enactment (Huang et al., 2018; Zhang et al., 2022). It is a rare diagnosis, with prevalence around 0.5%, with risk factors including prior pesticide exposure or head injury, lower educational level, male gender, increased age and prior psychiatric diagnosis (Gabryelska et al., 2018; Jung and Oh, 2021; Zhang et al., 2022). RBD can be secondary to medications or isolated RBD, and the latter is thought to be a precursor of alpha-synucleinopathies, such as Parksinson’s disease, pure autonomic failure, dementia with Lewy bodies (DLB), and multiple system atrophy. Thus, a diagnosis of RBD may be important due to its prognostic implications and may help alert the clinician of the likelihood of other possible symptoms that may occur with these conditions (Barone and Henchcliffe, 2018; Kaminska et al., 2018; Zhang et al., 2022). Furthermore, presence of RBD is one of the core diagnostic criteria for DLB (Iranzo and Santamaria, 2005).

Once referred to as idiopathic RBD, one multicenter study found a high rate of phenoconversion over time, creating the question of if isolated RBD may be a better term (Kaminska et al., 2018). Medication-induced RBD is postulated to be secondary to medications such as selective serotonin reuptake inhibitors and serotonin and norepinephrine reuptake inhibitors; although, whether the medication is a direct cause of RBD or leads to unmasking of early neurodegenerative disease is unknown (Simor et al., 2020). Furthermore, there are mixed data on this subject, as another cohort showed only evidence of REM sleep without atonia and not RBD in patients taking antidepressants. The long-term implications of RBD secondary to medications is controversial and unknown at this time (Manni et al., 2009).

Difficulties with blood pressure regulation and autonomic control have been described in patients with RBD, with orthostatic hypotension being the most common manifestation (Dzierzewski et al., 2018; McCarter et al., 2020; Zhang et al., 2022). Urinary retention and constipation are also relatively common symptoms seen in patients with RBD (Yaffe et al., 2011; Andrade et al., 2018). The mechanism for autonomic dysfunction in these patients is unknown but is thought to be due to the development of structural abnormalities in the areas of the brainstem that control autonomic function (Mansukhani et al., 2015; Zhang et al., 2022). A decreased hypocretin level leading to instability of the on/off function of the REM sleep centers in the brainstem has also been proposed (Zhang et al., 2022).

Patients with RBD without clinically evident neurodegenerative disorder at presentation receive a diagnosis of one of the previously mentioned neurodegenerative disorders at a rate of approximately 6% per year, with over 45–67.5% of patients developing an alpha-synucleinopathy within a decade of diagnosis (Barone and Henchcliffe, 2018; Gabryelska et al., 2018; Kaminska et al., 2018). One cohort showed that, of the patients who developed neurodegenerative disorders, the phenoconversion to PD occurred in 46.9% of patients, dementia with Lewey bodies in 46.2%, and multiple system atrophy in 6.9% (Yaffe et al., 2011). Patients with RBD have been shown to develop autonomic dysfunction years after the initial diagnosis, with the degree of autonomic dysfunction significantly progressing after the diagnosis of a neurodegenerative disorder (Barone and Henchcliffe, 2018; Zhang et al., 2022). Other studies have shown that patients can develop autonomic dysfunction and neurodegenerative disease symptoms in parallel (Barone and Henchcliffe, 2018). Another cohort found that change in color vision, olfactory and motor function, and not using antidepressants was also a risk factor for phenoconversion to a synucleinopathy (Jung and Oh, 2021). Gender also appears to play a role in patients with DLB, with men being more likely to develop RBD prior to cognitive dysfunction and women being more likely to develop RBD and cognitive dysfunction simultaneously (de Vivo et al., 2017).

Autonomic dysfunction in patients with RBD seems to be quite common, with one single center study showing an 80% prevalence (Barone and Henchcliffe, 2018). Patients who go on to develop dementia with Lewy bodies were also found to have more severe autonomic impairment than those who develop Parkinson’s disease (Barone and Henchcliffe, 2018). One study found that patients with DLB had longer sleep latency, shorter REM duration and lower sleep efficiency than those with isolated RBD. Patients with DLB also had a lower number of motor events during sleep (Peter-Derex et al., 2015). Interestingly, this study also found patients who were eventually diagnosed with dementia with Lewy bodies to have a longer duration of autonomic dysfunction prior to diagnosis compared to patients diagnosed with Parkinson’s disease (Barone and Henchcliffe, 2018).

Patients with Parkinson’s disease who do have RBD have been shown to have more severe autonomic dysfunction when compared to those who do not have RBD (Huang et al., 2018). In fact, there have been discussions of whether Parkinson’s disease with RBD (PD-RBD) is distinct from Parkinson’s disease without RBD (PD-nRBD) (Zhang et al., 2022). Studies have also shown that PD-RBD patients have more severe motor, cognitive, and psychiatric symptoms (Zhang et al., 2022).

Additionally, sleep apnea and RBD are common disorders found in patients with dementia, particularly in patients with Parkinson’s disease (Xie et al., 2013). As previously mentioned, the prevalence of RBD in Parkinson’s disease has been shown to be anywhere from 30 to 80% (Xie et al., 2013; Barone and Henchcliffe, 2018). OSA has also been shown to be common in this subset of patients, with a prevalence reported to be between 20 and 66% (Xie et al., 2013). OSA prevalence in patients with RBD has also been found to be quite high, with prevalence estimated between 34 and 89% (Lim et al., 2013; Hablitz et al., 2019). The link between OSA and RBD is not clear but is thought to be in part due to shared risk factors including older age and male gender (Lim et al., 2013). It has been hypothesized that patients with concomitant RBD and OSA may have greater cognitive dysfunction, with CPAP usage providing a potential benefit for RBD symptoms (Hablitz et al., 2019; Bubu et al., 2020).

Patients with severe sleep apnea have also been shown to experience abnormal nocturnal movements that may mimic RBD (Lim et al., 2013; Hablitz et al., 2019). These symptoms can include talking, gesturing, and punching during sleep (Lim et al., 2013). Symptoms associated with pseudo-RBD related to OSA usually occur during apnea-related arousals and can occur during both non-REM and REM sleep (Lim et al., 2013). Studies have shown that these pseudo-RDB movements are greatly decreased by use of CPAP therapy (Terzaghi et al., 2017). Therefore, this makes it difficult to assess if CPAP adherence improves RBD symptoms or simply concomitant pseudo-RBD movements (Hablitz et al., 2019).

Finally, REM sleep consists of both phasic and tonic REM. Phasic REM is characterized by bursts of eye movements, muscle twitches and saw-tooth waves on electroencephalography. Phasic REM also has greater irregularity in respiratory and cardiac output associated with an increased sympathetic drive. In contrast, tonic REM, consists of more quiet segments with predominant parasympathetic activity (Castel et al., 2020). RBD occurs much more frequently during phasic REM (Manni et al., 2009). These different states of REM sleep are associated with differential autonomic activity and future studies examining autonomic dysfunction in RBD should pay closer attention to these differences based on the specific state of REM sleep.

## Association between sleep apnea and dementia

Sleep and cognition have been shown to be closely linked, with older adults demonstrating a greater decrease in cognition when experiencing sleep deprivation or insomnia (Dzierzewski et al., 2018). In patients with OSA, decreases in memory and ability to perform executive functions have been described (Lutsey et al., 2018). In addition, OSA has been found to be associated with Alzheimer’s disease, although the exact mechanisms are unclear (Mansukhani et al., 2019). Patients with Alzheimer’s disease have been shown to have an increased prevalence of sleep apnea, with 40–70% of patients showing the presence of sleep disordered breathing, compared to 10-17% of the general male population (Mansukhani et al., 2019). Another study by Lutsey et al. (2018) showed that adult women with an AHI of over 15 events per hour were 85% more likely to develop mild cognitive impairment (MCI) or dementia. In the same study, patients with severe OSA were found to have an increased risk of all cause dementia and Alzheimer’s dementia with a risk ratio of 2.35 and 1.66, respectively (Lutsey et al., 2018). It has been postulated that the presence of sleep apnea could be a factor that perpetuates the emergence of dementia symptoms, possibly through hypoxia driven neuron dysfunction (Mansukhani et al., 2019). Ongoing hypoxemia has been shown to cause an inflammatory response, and thus endothelial dysfunction, with subsequent increased susceptibility to injury (Andrade et al., 2018; Culebras and Anwar, 2018). However, some studies have not shown an association between the degree of hypoxia experienced by patients with sleep apnea and subsequent risk of developing dementia (Yaffe et al., 2011). Other hypotheses include sleep fragmentation leading to increased emergence of cognitive dysfunction in patients with dementia (McCarter et al., 2020).

Sleep is an important function for restoration of synaptic function and memory consolidation and integration. A prospective study conducted in mice found that the axon-spine interface size decreased by approximately 18% during sleep, indicating renormalization of synaptic strength and thus consolidation and integration of neural input. This was more evident in small and medium synapses than in large synapses. These changes usually occur during slow wave sleep, which may be less in patients with dementia due to more frequent awakenings (de Vivo et al., 2017). Another study demonstrated that patients with multi-domain MCI experienced decreased sleep dependent memory consolidation when compared to controls and patients with single domain MCI. Patients with MCI had more frequent oxygen desaturation and less stage N2 sleep when compared to the control group (Lam et al., 2021).

A relationship between sleep disordered breathing and other causes of dementia has also been described. As previously mentioned, OSA can lead to sympathetic activation, and thus development of systemic hypertension. Systemic hypertension increases the risk of cerebrovascular accidents and thus, vascular dementia, which is the second most common cause of dementia in the United States (McCarter et al., 2020). Development of systemic hypertension in mid-life has been shown to substantially increase the risk of dementia, with childhood or early adulthood onset increasing the risk even further. Treatment of systemic hypertension has not been shown to decrease the risk of developing dementia, although the literature is mixed (Culebras and Anwar, 2018; McCarter et al., 2020). It has been hypothesized that OSA can theoretically worsen cognitive function through vascular lesions in the cerebrum (Peter-Derex et al., 2015). Furthermore, in patients with a stroke, untreated sleep apnea has been associated with increased cognitive decline (Culebras and Anwar, 2018).

While the associations between sleep apnea and Alzheimer’s disease have been most commonly been explored, there is also evidence that sleep apnea is common in other types of dementia. Frontotemporal dementia (FTD) is characterized by degeneration in the frontal and temporal lobe and the disease process in this condition can impact sleep related circuitry in the hypothalamus and basal for brain. Patients with FTD have higher rates of sleep disordered breathing as compared to healthy controls. Further, autonomic dysfunction is also thought to occur in patients with FTD but there are no current studies examining the role of sleep apnea in mediating autonomic dysfunction in FTD.

While the exact functions of sleep are still under debate, a more recent theory proposes that sleep plays an important role in clearing toxins that accumulate during the day. Initial studies demonstrated that there is an increase in cerebrospinal fluid tracer activity that does not occur in wakefulness but occurs after the onset of sleep or induction of anesthesia, through the glymphatic system (Bonakis et al., 2014). More recent studies have demonstrated that this is likely to occur with increases in slow-wave activity (Ahmed et al., 2015). Amyloid beta, a precursor of the plaques implicated in Alzheimer’s disease, are cleared by the glymphatic system. Sleep fragmentation, which is a common consequence of sleep apnea, can decrease the amount of slow-wave sleep activity and may thus increase the risk of developing AD (Xie et al., 2013).

As previously reported, the exact mechanism underlying the relationship between dementia and sleep disordered breathing remains unclear. There are data to suggest, however, that treatment of sleep apnea can help alleviate the symptoms of dementia. Patients who are adherent to CPAP therapy have been shown to have greater improvement in cognitive processing speed as well as other sleep parameters (Hablitz et al., 2019). A more recent study also showed decreased odds of developing MCI or Alzheimer’s disease in Medicare beneficiaries with OSA that were adherent to CPAP treatment (Peter-Derex et al., 2015). Another recent study of patients with Parkinson’s disease reported that CPAP usage improved non-motor symptoms such as sleep quality, cognitive function, and anxiety (Bubu et al., 2020). Complicating the issue, CPAP tolerance has been shown to be problematic inpatients with Parkinson’s disease. One single center study showed a 75% intolerance rate while another study showed CPAP intolerance in 50% of individuals, necessitating another treatment strategy (Lim et al., 2013; Bubu et al., 2020). The data on this complex subject continue to remain mixed, however (Huang et al., 2018; McCarter et al., 2020).

## Conclusion

Sleep apnea is a common diagnosis made in the general population, with prevalence increasing over the years. Sleep apnea, autonomic dysfunction, rapid eye movement sleep behavior disorder, and dementia are all commonly associated with each other and closely linked. Sleep apnea is associated with autonomic dysfunction. Autonomic dysfunction is common in patients with rapid eye movement sleep behavior disorder (which can be a precursor of dementia with Lewy bodies and other alpha-synucleinopathies) and dementia. Finally, sleep apnea is associated with various types of dementia.

In this article, we have provided a review of the autonomic changes seen in subjects with sleep apnea. We also describe the autonomic dysfunction seen in patients with rapid eye movement sleep behavior disorder and dementia. While the relationship between sleep apnea and autonomic dysfunction is well documented, the link between autonomic dysfunction and rapid eye movement sleep behavior disorder and dementia requires further study. Currently, there appears to be relationship between sleep apnea the development of dementia; and it may be possible that untreated sleep apnea may be a risk factor for the earlier onset of cognitive dysfunction due to accompanying intermittent hypoxia and sleep fragmentation. The pathophysiologic link between sleep apnea and dementia, especially we associations between sleep apnea, autonomic dysfunction and their role in the development of dementia merits further study. Furthermore, whether patients with significant sleep disordered breathing should be monitored for changes in autonomic function as well as cognitive decline is an area that needs further investigation. Similarly, research on the subpopulations of patients with sleep apnea that would require treatment to prevent the onset of cognitive dysfunction, as well as the best tools to screen for such dysfunction, and head-to-head comparisons of the various treatment options for sleep apnea on patient-reported and other long-term outcomes in patients with and without dementia is needed. With the increasing prevalence of sleep apnea across all age ranges, the relationship between sleep apnea and the aforementioned disorders would benefit from further investigation into the underlying pathophysiology as well as the impact of treatment on long-term patient-reported outcomes and quality of life.

## Author contributions

MH wrote the manuscript. BK edited the manuscript. MM edited the manuscript and provided supervision of the project. TP and PM provided critical review of the manuscript. All authors contributed to the article and approved the submitted version.

## Abbreviations

REM: rapid eye movement
RBD: rapid eye movement sleep behavior disorder
OSA: obstructive sleep apnea
CPAP: continuous positive airway pressure
PD-RBD: Parkinson’s disease with REM sleep behavior disorder
PD-nRBD: Parkinson’s disease without REM sleep behavior disorder.
